# Modifications on Promoting the Proton Conductivity of Polybenzimidazole-Based Polymer Electrolyte Membranes in Fuel Cells

**DOI:** 10.3390/membranes11110826

**Published:** 2021-10-27

**Authors:** Junyu Chen, Jiamu Cao, Rongji Zhang, Jing Zhou, Shimin Wang, Xu Liu, Tinghe Zhang, Xinyuan Tao, Yufeng Zhang

**Affiliations:** 1 School of Astronautics, Harbin Institute of Technology, Harbin 150001, China; cjy9291@126.com (J.C.); 15663592622@163.com (R.Z.); daxiongmao@hit.edu.cn (J.Z.); 18800421178@163.com (S.W.); liuxu21S0416@163.com (X.L.); nbiryhct@126.com (T.Z.); 1182100225@stu.hit.edu.cn (X.T.); yufeng_zhang@hit.edu.cn (Y.Z.); 2 Harbin Institute of Technology, Harbin Inst Technol, Res Ctr Space Opt Engn, Harbin 150001, China; 3 Key Laboratory of Micro-Systems and Micro-Structures Manufacturing, Ministry of Education, Harbin 150001, China

**Keywords:** polybenzimidazole, polymer electrolyte membrane, proton exchange membrane, PEMFC, DMFC, proton conductivity

## Abstract

Hydrogen-air proton exchange membrane fuel cells (PEMFCs) and direct methanol fuel cells (DMFCs) are excellent fuel cells with high limits of energy density. However, the low carbon monoxide (CO) tolerance of the Pt electrode catalyst in hydrogen-air PEMFCs and methanol permanent in DMFCs greatly hindered their extensive use. Applying polybenzimidazole (PBI) membranes can avoid these problems. The high thermal stability allows PBI membranes to work at elevated temperatures when the CO tolerance can be significantly improved; the excellent methanol resistance also makes it suitable for DMFCs. However, the poor proton conductivity of pristine PBI makes it hard to be directly applied in fuel cells. In the past decades, researchers have made great efforts to promote the proton conductivity of PBI membranes, and various effective modification methods have been proposed. To provide engineers and researchers with a basis to further promote the properties of fuel cells with PBI membranes, this paper reviews critical researches on the modification of PBI membranes in both hydrogen-air PEMFCs and DMFCs aiming at promoting the proton conductivity. The modification methods have been classified and the obtained properties have been included. A guide for designing modifications on PBI membranes for high-performance fuel cells is provided.

## 1. Introduction

Fossil energy which is non-renewable still plays the dominant role in various energy applications [1,2]. However, with the increase in energy demands and the dwindling reserves of fossil energy, it has become more and more urgent to find alternative energy or conversion technologies [3]. The environmental degradation brought by the burning of fossil fuels further stressed the necessity. In recent decades, fuel cells, as an energy conversion technology, have received lots of attention for their considerable energy conversion efficiency and for being environmentally friendly. They have great potential in performing as dominant power sources in various applications [4,5]. Moreover, different suitable energy sources that have high energy densities have been adopted in fuel cells, such as hydrogen, methanol, and ammonia [6]. Among them, hydrogen has the highest energy density, and fuel cells using hydrogen energy are usually hydrogen-air proton exchange membrane fuel cells (PEMFCs). While the problem of hydrogen storage obstructed its extensive use. Though the problem could be solved by in-situ hydrogen generation methods such as methanol-reforming, the accompanying CO gas can poison the electrode catalyst and make it invalid [7]. Methanol also has a relatively high energy density and can be applied in direct methanol fuel cells (DMFCs). However, the problem of methanol crossover in DMFCs also hindered its development [8]. To solve the problems in PEMFCs and DMFCs, great efforts have been made [9,10,11,12]. Our previous work has also reviewed the critical investigations of methanol-resistant PEMs in DMFCs [13].

The problems mainly derive from the polymer electrolyte membranes (PEMs) part where protons pass through to finish the process of proton exchange from anode to cathode. In hydrogen-air PEMFCs, if CO molecules can be adsorbed more easily on the Pt hydrogen oxidation catalyst of the membrane electrode assembly (MEA) than H_2_ molecules, the active sites for hydrogen oxidation are occupied, then protons cannot be generated and the cell reaction stopped [7]. In DMFCs, if a great number of methanol molecules can also pass through the PEM, methanol crossover occurs and would bring various problems [9,12]. It is mentionable that polybenzimidazole (PBI) membranes, a family of aromatic polymer membranes with benzimidazole units, have great potential in solving both the problems in PEMFCs and DMFCs [14]. For hydrogen-air PEMFCs, especially those integrated with reformers, applying PBI-based membranes could greatly improve the CO tolerance, from 10 ppm for traditional membranes that work at low temperatures to about 2% [15]. For DMFCs, using PBI-based membranes could greatly reduce the methanol permeability [16], as pure PBI membranes have extremely low methanol permeability (about 5 × 10^−9^ S cm^−1^) [17]. However, the proton conductivity of the PBI membrane is also low, which will greatly reduce the performance of fuel cells and makes it difficult for fuel cells using PBI-based membranes to compete with traditional PEMs (such as Nafion membranes) in performance [18]. To promote the proton conductivity of PBI membranes without sacrificing the chemical stability and the mechanical strength, various attempts have been applied, such as doping with acid [19,20,21], synthesizing modified PBI-based structures [22,23,24], applying nanomaterials [25,26,27], combining other cross-linkers [28,29,30] and building porous microstructures [31,32,33]. Each of the methods has achieved remarkable promotions.

Though the final goal of all these methods is promoting the proton conductivity of PBI-based membranes, the mechanisms are different. For the methods of building porous microstructure membranes and synthesizing modified PBI-based structures, they are aiming at modifying the nature of the PBI membrane itself. While other methods are making efforts on borrowing superior proton conductivity characters from other materials, then combining that with PBI. Methods modifying the proton conductivity properties of PBI membrane can also influence other properties that are not expected to change [34,35]; methods combining other materials could also bring adverse effects. Besides, the key issues of the application of PBI-based membranes in hydrogen-air PEMFCs and DMFCs are also different [36,37]. The inferior proton conductivity of PBI-based membranes makes them not competitive in room temperature hydrogen-air PEMFCs [38], while the high thermal stability and higher CO tolerance make them irreplaceable in high-temperature PEMFCs (HT-PEMFCs). Thus, the modifying of PBI-based membranes in hydrogen-air PEMFCs cannot sacrifice thermal stability. For DMFCs, the excellent methanol resistance is the most valuable for PBI-based membranes, it should not be ignored in the process of modifying. These issues make the promoting of the proton conductivity of PBI-based membranes more difficult, and researches have not been terminated till now.

Intensive works have been published in promoting the proton conductivity of PBI-based membranes in fuel cells. Though most of the methods are still at the experiment stage and far from applications, they are promising and have the potential to make PBI-based membranes irreplaceable in corresponding fuel cell applications. Inspired by this, a sufficient survey on the studies in modifying PBI-based membranes is necessary. Thus, in this review, recently reported methods to promote the proton conductivity of PBI-based membranes in both hydrogen-air PEMFCs and DMFCs are classified and summarized. Aiming at providing a guide for designing modifications on PBI membranes for high-performance fuel cells. Section 3 describes the progress in modifying the PBI-based membranes in hydrogen-air PEMFCs. And in Section 4, methods of promoting the proton conductivity of PBI-based membranes in DMFCs are presented. Before showing these, the following Section 2 explains the mechanisms of the high CO tolerance of PBI-based membranes in hydrogen-air PEMFCs and the high methanol resistance of PBI-based membranes in DMFCs. Besides, the principles of different categories of methods to promote the proton conductivity of PBI are also demonstrated in that section.

## 2. General Aspects of PBI-Based Polymer Electrolyte Membranes

MEAs with PBI-based membranes usually have high CO tolerance in hydrogen-air PEMFCs and high methanol resistance in DMFCs. Although, what character of PBI achieved these properties? The detailed mechanisms are presented in this part. Moreover, PBI also has poor proton conductivity, in what way can give promotion on this? The available modification mechanisms are also addressed in this part.

### 2.1. Mechanism of CO Tolerance in HT-PEMFCs

**Figure 1:**
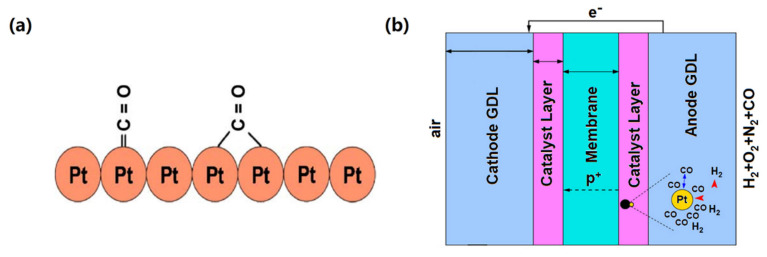
Sketch Map of (**a**) CO on Pt surface from Ref. [7] and (**b**) Anode catalyst layer in PEMFCs from Ref. [41].

The performance of PEMs for hydrogen-air fuel cells is usually tested under pure hydrogen. However, in practical applications, pure hydrogen is hard to obtain and difficult to store. By comparison, hydrogen-rich gases are closer to reality as fuels, they can be efficiently produced on-site by reforming reactions [39,40]. While the reformate gases usually contain carbon monoxide (CO) that could be strongly adsorbed on Pt surfaces and occupy the active sites of hydrogen oxidation reaction (HOR), which has a significant impact on the electrode reaction [7,41], as presented in Figure 1.

An effective way to solve the problem is to alleviate CO adsorption on the catalyst. As the adsorption of CO on Pt has high negative entropy, indicating it is prone to occur at low temperatures. While if the temperature becomes higher, it can be restricted. For instance, Pt-based catalysts can bear 10 to 20 ppm CO at 80 °C, and up to 1000 ppm CO at 130 °C [42], and higher CO concentration at higher working temperatures (30,000 ppm at 200 °C) [43]. With this, the problem shifts to finding PEMs that can work at higher temperatures (above 100 °C). Though the temperature from 100 to 200 °C still belongs to low temperature in the engineering sense, it would bring a great challenge to PEMFCs that usually work at a temperature below 80 °C [14]. First, the elevated temperatures will give ordeals to the chemical and mechanical stabilities of PEMs. Second, in temperatures above 100 ℃, maintaining the humidity of MEAs by water steam will greatly improve the pressure and significantly increase the complexity of the design of fuel cell systems. To solve these problems, great efforts have been made. The excellent thermal stability of PBI (glass transition temperature at 425–436 °C) makes it outstanding in such applications [14]. A large number of works on designing PBI-based membranes working at a temperature range of 100–200 °C have been reported [42,43,44].

### 2.2. Mechanism of Methanol Crossover and Methanol Resistance in DMFCs

It is well known that methanol crossover in the PEMs greatly obstructs the extensive use of DMFCs, especially those with Nafion PEMs. Methanol penetrated through the membrane can also be oxidized at the cathode, which would result in the formation of mixed potential at cathode catalyst particles [45]. Besides, the oxidation products of methanol can also poison the cathode catalysts [46]. Modifications should be made on PEMs to solve the problem. Thus, understanding the mechanism of methanol crossover is critical before taking measures, and attention should be paid to methanol resistance strategies.

**Figure 2:**
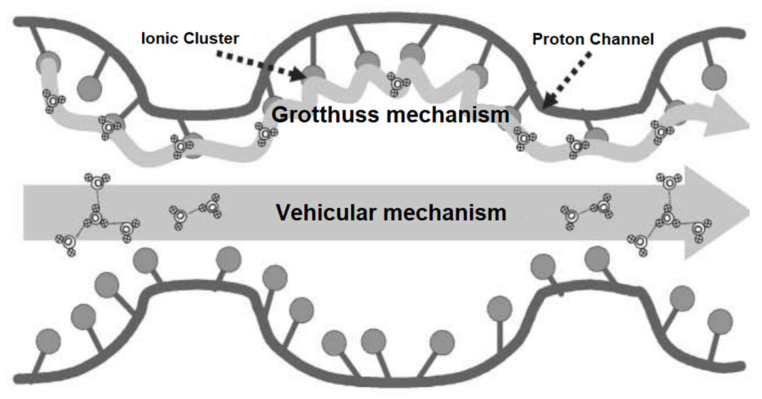
Schematic diagram of protons transport in PEM when DMFC is in operation. Reproduced from Ref. [47].

In the working process of DMFCs, the transportation of protons relies on free water molecules in the micropores of the membrane [47]. Protons can exist in the form of H_3_O^+^ in water and be transported through the PEM by electrochemical difference diffusion, this is the Vehicular mechanism [48]. Unfortunately, methanol molecules can also flow through the DMFC under the electric dragging effect of hydrating protons. Such effect is considered to be the dominant mechanism of methanol crossover. Besides, protons can also transport in another mechanism (Grotthuss mechanism). In that transport process, protons bind with H_3_O^+^ groups are attached to ionic clusters (such as sulfonic acid groups). After that, one proton in H_3_O^+^ moved to the water molecule combined with an adjacent ionic cluster. In the way of breaking and establishing hydrogen bonds, protons could transport through the PEM by hopping on ionic clusters. In this mechanism, protons are considered to transport faster [49]. The two proton transport mechanisms are demonstrated in Figure 2, both the proton transport mechanisms are considered to be active in a working DMFC. However, as the main mechanism of the solution transport in membranes is electric dragging, preventing methanol permeation is nearly impossible for Nafion PEMs. Methanol crossover can be reduced in low humidity conditions, while the application range is also restricted. Applying non-Nafion PEMs can also reduce methanol permeation, but the proton transport is usually restricted as well.

To evaluate the overall methanol resistance of PEMs, membrane selectivity is usually used. It can be calculated by the ratio between the proton conductivity to the methanol permeability. A high value of membrane selectivity indicates excellent overall performance. It is well known that PBI-based PEMs have excellent methanol resistance. For PBI membranes, the selectivity of the proton is higher than other traditional PEMs (such as Nafion-based membranes) [13], indicating methanol molecules are hard to be dragged through PBI membranes with the water flow. Such character can be determined by the membrane morphology. Moreover, the Grotthus mechanism has also been proven to be active in the proton transport of PBI membranes [50].

Another important parameter to evaluate the properties of membranes is durability. As the chemical and mechanical properties of the membrane degrade with the increase in working hours in the harsh environment of the fuel cell, there is a limit for the maximum working hours of membranes. Including that methanol, crossover might be more serious when the membrane is close to the limit. Such an index is always in great concern in actual fuel cell applications.

### 2.3. Methods to Optimize the Proton Conduction in PBI-Based PEMs

To make PBI membranes more competitive in fuel cell applications, the proton conductivity should be significantly improved. As mentioned in Section 1, methods to promote the proton conduction of PBI membranes could be classified as modifying the nature of PBI and combining them with other materials with excellent proton conductivity. Besides, the target to promote the proton conduction without changing the stability and methanol resistance nature of PBI membranes makes the methods more complex.

Among all the methods, doping with acid is the most basic one and most modification methods are based on that. The unique proton conductivity and excellent thermal stability of phosphoric acid make it most concerned in modifying PBI membranes [14,51]. Besides, phenyl phosphonic acids and organophosphonic acids are also applied to achieve better acid retaining properties [14]. When phosphoric acid doped, the formed H_2_PO_4_^−^ anion brought strong hydrogen bonds and pushed the process to form an infinite network with structures of organic cationic monophosphates [52,53,54,55]. With this, the proton transport in H_3_PO_4_/PBI mainly through the hopping (Grotthus) mechanism that has high efficiency [56]. For PBI membranes, a higher acid doping level usually means greater promotion of proton conductivity.

Adding additional groups and modifying the main chain of polymers are common in functionalizing materials. That means adjusting the formula and the process parameters of synthesis. For the modification of PBI, various groups have been applied, such as sulphone, aliphatic, ether, and ketone. The added groups can help to hold the polymer molecules together and increase the molecular weight. Thus, the acid-doping level can be further improved and the selectivity of the hopping transport mechanism is promoted.

Applying nanomaterials is also effective in modifying the character of materials. Among them, forming inorganic-organic composites is the most popular. With a solid proton conductor (such as Zr(HPO_4_)_2_) added, the proton conductivity of the synthesized membrane can be directly increased. Otherwise, adding graphene-based materials can also achieve similar effects as effective pathways for proton transfer are formed through the interaction between fillers and polymer matrix [57,58].

Another promising method is combing PBI with other polymer crosslinkers. The applied crosslinkers can be either ionic bonded or covalently bonded with PBI. With the formed flexible networks, the acid doping level of PBI-based membranes can be further improved so that the proton conductivity is improved [59,60]. Besides, the thermal stability is usually enhanced by covalent crosslinkers and the toughness of membranes is better for ionic-crosslinked ones.

Modifying the morphology of membranes is also promising that cannot be ignored. With porous structures, the phosphoric acid can fill the mesopores and the interfacial energy is reduced, it may also chemically bond with benzimidazole moieties [61]. With these effects, the acid adsorption can be improved and the leakage is reduced [32,61,62]. With the improved carrying capacity of acid, the proton conductivity of porous PBI-based membrane is sure to be higher.

These methods can also be flexibly combined and achieve much superior proton conductivity in PBI-based membranes, especially in recent researches [63].

## 3. Progress of Modification of PBI-Based Membranes for Hydrogen-Air PEMFCs

### 3.1. Modification of PBI Membranes for Hydrogen-Air PEMFCs by Acid Doping

The proton conductivity of PBI membranes needs to be significantly improved for fuel cell applications. Acid doping is the most common method to achieve this goal, and all the following introduced methods are based on acid-doped PBI membranes. As PBI membranes usually work at a high temperature (above 120 °C), the applied acid is also required to have good thermal stability. Among many inorganic acids, phosphoric acid becomes outstanding as the excellent thermal stability and unique proton conductivity under anhydrous conditions [64]. Most studies on acid modification of PBI membranes for fuel cells are focused on phosphoric acid and aim at improving acid-doping levels (mole numbers of H_3_PO_4_ per PBI mole). Table 1 presents several representative studies on promoting the proton conductivity of PBI PEMs in hydrogen-air PEMFCs by acid doping.

Doping phosphoric acid to PBI was developed in the late 20th century. The aim of doping can be achieved by immersing PBI membranes in an aqueous acid solution. While the acid doping level is usually low by this means. It can only reach 5–6 without ignoring the mechanical strength of the membrane [14]. For instance, Lobato et al. [65] used the casting method of phosphoric acid immersing and got an acid doping level of 6.2. They found that the stress at break of the PBI membrane with an acid doping level over 5 is only one-tenth of that for the PBI membrane with an acid doping level of 2. Restricted by this, the casted membranes were tested to have a proton conductivity of 0.039 S cm^−1^ at 60 °C which is relatively low compared with PBI membranes today. To give improvements on this, trifluoroacetic acid has been applied and mixed with phosphoric acid as a soaking solution. With this method, the acid doping level can be raised to a higher level. Besides, by applying nanofillers or crosslinkers, the phosphoric acid doping level in PBI can be further improved (to more than 20) [66], more details are introduced in the next section.

Besides phosphoric acid, other acids, such as phenyl phosphonic acids, alkyl phosphonic acids, and phosphotungstic acids have also been applied to improve the acid retaining properties and mechanical strength of PBI membranes [67]. For instance, He et al. [68] applied phosphotungstic acid (PWA) and silicotungstic acid (SiWA) to PBI for improving the proton conductivity. Authors found that by introducing PWA and SiWA, the PBI membranes own better mechanical strength than simple PA doped PBI, the proton conductivity is also comparable with that of PBI membranes with a PA doping level of 5.6. The main reason for the improved proton conductivity is the high intrinsic proton conductivity of the crystalline forms of the hydrated heteropoly acids.

However, with the increase in working hours, the doped acid would be released which will significantly decrease the proton transport properties of PBI membranes [26]. To compensate for this, many modification methods have been designed. Such as adding phosphonates and sulfonate groups. As reported by Suryani et al. [69], the added sulfonic acid groups in the PBI chain can form covalent bonds with acid groups which have great benefits to acid retaining. With this method, they got a phosphoric acid doping level of 12.3 and much higher proton conductivity than simple PA-doped PBI. But it is still hard to completely avoid the acid leakage effect. The working life of acid-doped PBI membranes will be greatly improved if the problem can be solved.

### 3.2. Modification of PBI Membranes for Hydrogen-Air PEMFCs by Synthesizing Modified Structures

To improve the proton conductivity of PBI membranes, methods of synthesizing modified structures of PBI polymer are commonly applied. Such methods have been studied from more than 40 years ago [76,77], and are still of concern recently [78,79]. The specific methods can be divided into modifying the main chains of PBI and post-polymerization functional groups, and they can also be combined.

**Figure 3:**
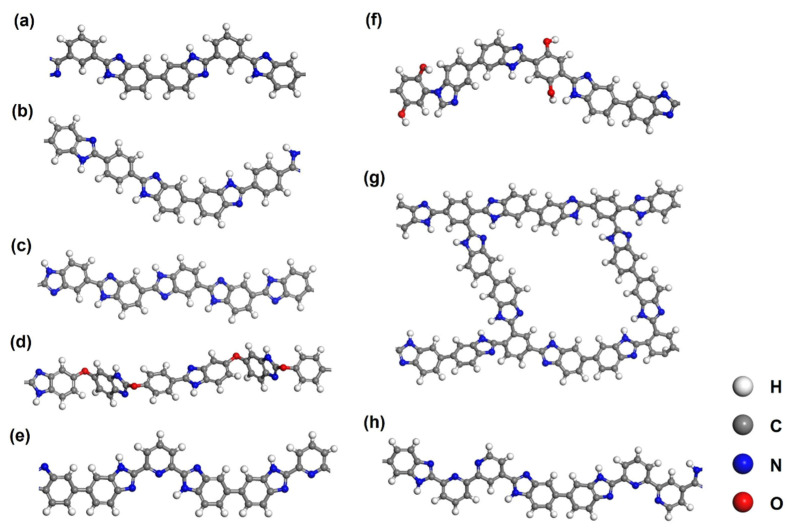
Graphs of polymer structures for (**a**) m-PBI, (**b**) para-PBI, (**c**) AB-PBI, (**d**) OOPBI, (**e**) Py-PBI, (**f**) 2OH-PBI, (**g**) HB-PBI, and (**h**) Bipy-PBI.

An array of methods to modify the main chain of PBI have been reported, such as para PBI(p-PBI) [80], AB-PBI [81], Py-PBI [14] and OO-PBI [78]. The polymer unit structures are shown in Figure 3b–e. Most of them have shown promoted proton conductivity to pristine PBI (m-PBI). For instance, early in 2009, Yu et al. [82] prepared PEM with 2OH- modified PBI and doped with polyphosphoric acid (PPA). They got an extremely high proton conductivity of 0.35 S cm^−1^ at 160 °C as the high acid doping level of 25.4. The authors revealed that with the additional -OH groups, the doped phosphoric acid can react with them and form ester linkages, the polymer structure of 2OH-PBI is presented in Figure 3f. With this effect, the acid doping levels can be much higher than that for simple m-PBI, and thus greatly raise the proton conductivity. Liu et al. [83] synthesized a kind of hyper-branched PBI (HB-PBI). As presented in Figure 3g, the polymer unit of HB-PBI amounts to nearly three m-PBI units that can offer more sites to adsorb acid groups. Similarly, they also found HB-PBI can accommodate high acid doping levels that result in high proton conductivities of membranes. Lin et al. [84] prepared butyl sulfonate grafted PBI (PBI-BS) blends and applied them in PEMFC. With the butyl sulfonate groups on PBI, the chains of polymers can link together and form polymers with high molecule weights (more than 9.16 × 10^4^). With this, the ability to accommodate PA groups becomes stronger. The prepared membrane exhibit a proton conductivity of 31.2 mS cm^−1^ at 160 °C with considerable mechanical strength, and the maximum fuel cell power density reached 551 mW cm^−2^. Lately, Mohamed et al. [79] synthesized a kind of phenylene-based PBI (Bipy-PBI) which has two more nitrogen atoms available for acid doping, as presented in Figure 3h. They got much higher proton conductivity than the original m-PBI as the high acid doping level. Besides, they also prepared Bipy-PBI polymers with different molecule weights. Authors found that groups with higher molecule weight possess higher proton conductivity for their higher degree of crosslinking that can bring more acid adsorption sites. With the optimized Bipy-PBI membrane, a high fuel cell power density of 779 mW cm^−2^ was obtained.

Post polymerization is also an effective method according to previous works. Such methods were developed because of the chemically reactive character of NH groups in the imidazole rings. Methods of adding groups (such as alkyl, phenyl, cyanoethyl, sulphoalkyl, and hydroxyethyl [14]) to the N sites of PBI units [85] have been designed and achieved improved properties in the conductivity of PEMs. While in recent years, such modifications are seldom mentioned because of their inferior performance compared with other modified PBI membranes.

### 3.3. Modification of PBI Membranes for Hydrogen-Air PEMFCs by Nanomaterials

As introduced in the part of acid doped PBI membranes, doping acids (phosphoric acid) can efficiently improve the proton conductivity of PBI membranes but the doping level is limited by the acid retention capacity and mechanical strength of membranes [14,86]. Such problems make acid-doped PBI membranes still hard to apply in fuel cell applications. Synthesizing modified structures could help, but is still limited in acid retaining. Aiming at seeking other solutions for the problem, more and more studies have attempted to apply inorganic nanomaterials to PBI membranes to fabricate organic-inorganic nanocomposites in recent years. With organic-inorganic composites, the acid doping level can be raised to a higher level and the proton transport in PBI membranes become more inclined to the Grotthuss mechanism that has high efficiency, especially for anhydrous conditions [87,88]. In the progress of the development of organic-inorganic composites, SiO_2_ is a good candidate that is commonly used. Such as Ghosh et al. [66] added amine-modified silicon dioxide (AMS) into poly (4,4′-diphenylether-5,5′-bibenzimidazole) (OPBI) matrix and fabricated OPBI/AMS composite membrane. The proton conductivity and PA doping level of the nanocomposite hybrid membrane are much higher than that of the pristine OPBI membrane and can increase with an increase in the nanofiller content. The authors explained that in PA media, silica nanoparticles that act as alkali can absorb phosphoric acid, which may contribute to doping more PA molecules in nanocomposite membranes. In addition, the presence of AMS nanoparticles and their self-assembled clusters promote proton transport better than that with unmodified silica nanoparticles which have more dispersed particles. The combined action of these two can make significant improvements in the proton conductivity. Similarly, Chu et al. [26] also applied Silica nanoparticles to synthesize PBI membranes and combined that with a zwitterion coat. They tested the proton conductivity of the synthesized membrane at anhydrous conditions and got a high proton conductivity of 10^−2^ S cm^−1^ at 160 °C. Authors proved that the zwitterion-coated SiO_2_ nanoparticles can prevent the release of phosphoric acid effectively. The chemical stability of the PBI membrane was also slightly improved by this method and more suitable to be applied in HT-PEMFCs.

**Figure 4:**
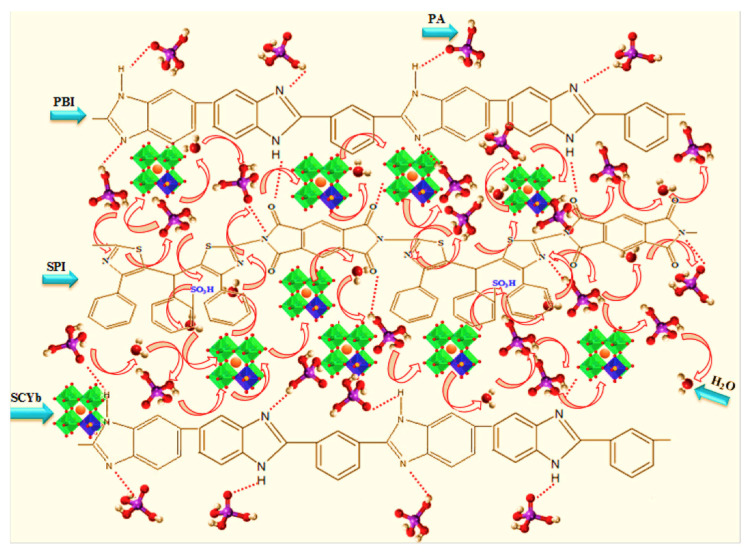
Scheme of the proton transport mechanism for PPBI-SPI_25_-SCYb_7_ nanocomposite membranes from Ref. [63].

In addition to SiO_2_ nanomaterials, many other inorganic nanomaterials have been applied. For instance, Hooshyari et al. [63] applied doped-perovskite nanoparticles and a new synthesized sulfonated polyimide (SPI) to PBI and fabricated SPI/PBI/SCYb nanocomposite blend membranes. It is found that the synthesized membrane can accommodate a high acid doping level of 14 (mol phosphoric acid per monomer unit), and showed a high proton conductivity of 131 mS cm^−1^ at 180 °C. With such membranes, a high power density of 0.59 Wcm^−2^ at 0.5 V was obtained. Authors attributed the higher proton conductivity performance to the substitution of Ce^4+^ by Yb^3+^ in doped perovskite nanoparticles which can generate oxygen vacancies and reduce coulombic repulsion between protons and positive ions, as presented in Figure 4.

### 3.4. Modification of PBI Membranes for Hydrogen-Air PEMFCs by Polymer Crosslinkers

Other than applying nanomaterials, crosslinking is also an effective way to improve the proton transport properties of PBI PEMs in hydrogen-air fuel cells. Similar to adding nanomaterials, such modifications in PBI mainly manifest in promoting the acid doping levels. Many kinds of polymer crosslinkers have been applied, such as diglycidyl epoxy resin (TMBP) [28], ferric sulfophenyl phosphate (FeSPP) [89], chloromethylation aromatic polymers [90], sulfonated polyimide (SPI) [63], etc. With the additional polymer crosslinkers, more active groups to accommodate phosphoric acid groups appeared, and the acid doping level can be much higher than m-PBI. In addition, the selected crosslinkers usually can improve the mechanical or chemical stability of the synthesized membranes.

**Figure 5:**
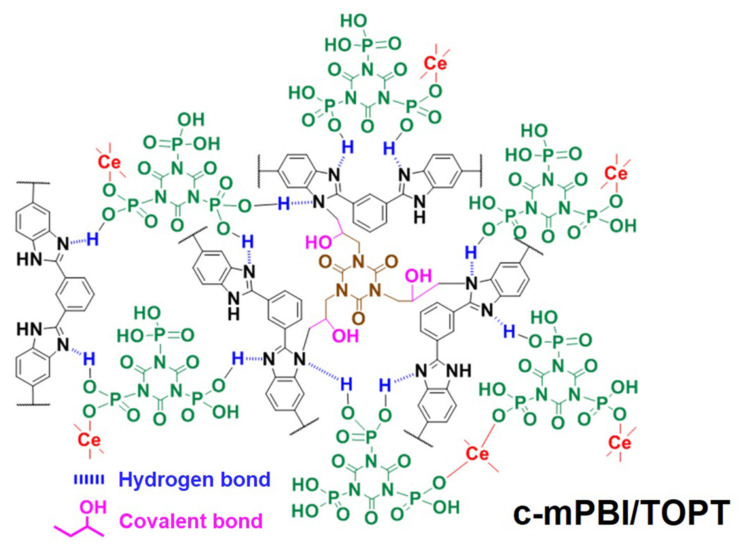
Chemical structure of c-mPBI/CeTOPT composite membrane from Ref. [92].

Crosslinking can be either ionic bonded or covalently bonded, both can improve the proton conductivity of PBI PEMs. As introduced in Section 2, PBI PEMs with ionic bonded crosslinkers usually have good toughness. For instance, Liu et al. [91] prepared a novel HT-PEM based on norbornene PBI (NbPBI) with poly (ionic liquid) PIL. A cross-linked network can be formed by the reaction of PILs with the norbornene monomer of NbPBI. Moreover, the Br^−^ and Cl^−^ of the PILs are reported to have replaced with H_2_PO_4_^−^ and thus improved the acid doping level of the composite membrane. Finally, a high proton conductivity of 0.074 Scm^−1^ at 170 °C was obtained, which is higher than NbPBI (0.056 S cm^−1^ at 170 °C). Organic-inorganic compositions have also been applied to form crosslinking structures, such as Guo et al. [92] incorporated cerium triphosphonic-isocyanurate (Ce-TOPT) into the PBI membrane and constructed HT-PEMs with -PO_3_H_2_ groups. The proton conductivity of the prepared membrane reached 0.125 S cm^−1^ at 180 °C. The authors presented that the introduction of TGIC crosslinker significantly add to the Grotthus mechanism of proton transport at low humidity. As in Ce-TOPT, a large number of hydrogen bond donors and acceptors can form a continuous hydrogen bond network in the membrane, realizing easier proton transfer at high temperature and low humidity, details are shown in Figure 5.

PBI PEMs with covalently bonded crosslinkers usually have excellent thermal stability, as reported by Sun et al. [29]. Authors synthesized PEM with covalently crosslinked triglycidylisocyanurate (TGIC) and PBI. In addition, highly sulfonated polyaniline (SPAN) was doped for further modification. The synthesized PBI-TGIC(5%)/SPAN(50%) PEM exhibits very high proton conductivity of 0.13 S cm^−1^ at 180 °C, which is much higher than the PA-PBI PEM reported in their previous work [93,94]. The promotion of proton conductivity is mainly attributed to the low crosslinking degree of TGIC, which allows a high doping level of SPAN. With more SPAN doped, more hydrogen bonding networks could be formed which greatly add to the PA retaining properties [95]. Besides, the covalently crosslinked structure also adds to the thermal stability and oxidative resistance properties of the synthesized PEM.

Moreover, different preparation methods have been applied to synthesize crosslinked structures. Besides solvent casting, methods of hot pressing [89], electrospinning [96] have also been adopted. Hot pressing methods are usually used to fabricate hard and brittle materials; electrospinning methods are usually for creating fine fibers that can play key roles in proton transport. As reported by Li et al. [96], the electrospinning process was applied to synthesize polybenzoxazine (PBz)-modified PBI nanofibers. The crosslinked PBI nanofibers have shown excellent mechanical strength and the synthesized composite membrane presented a high proton conductivity of 0.17 S cm^−1^ at 160 °C. Authors attributed the high performance to the orientation of the ion-conducting domains in the nanofibers and the formation of long-range ionic channels along the nanofibers.

### 3.5. Modification of PBI Membranes for Hydrogen-Air PEMFCs by Designing Porous Structures or Metal-Organic Frames

Another effective way to promote the proton conductivity of PBI membranes is to prepare porous structural materials. It has been reported that the acid doping level can be greatly improved by porous structures as the disconnected pores can be filled with PA [33]. Many methods have been applied to create porous PBI structures, such as doping with ionic liquid [97] or synthesizing pore-filled membranes [98]. For instance, Ven et al. [94] applied the method of doping ionic liquid. A volume porosity of about 65.6% was obtained for ionic liquid doped PBI (PBI/IL). With the porous structure, PA groups can be better accommodated and improve the acid doping level. Kim et al. [98] synthesized a reinforced porous pore-filling membrane based on benzoxazine-benzimidazole copolymers. The authors mentioned that the porous substrate should have a large pore size and be porosity. With these characters, the largest amount of proton-conducting ionomer can be contained and added to the membrane conductivity.

Moreover, the proton conductivity of PBI membranes can also be improved by designing metal-organic frameworks (MOF) structures. Zeolitic imidazolate frameworks (ZIFs) are common MOFs that have been used in various areas. For PA-doped PBI membranes, ZIFs can promote the Grottuss mechanism of proton transport by offering a network of hydrogen bonds [99], resulting in high efficiency of proton conductivity. Escorihuela et al. [57] chose to embed ZIF particles into polymer PBI matrix, which is a subclass of MOF. As they reported, the hybrid PBI@ZIF-mix membrane exhibited high proton conductivity at 200 °C, with a maximum value of (9.1 ± 0.2) × 10^−2^ S·cm^−1^. The proton transport by the Grotthuss mechanism [100] in PBI membranes is greatly promoted by the embedded ZIF MOFs. Authors analyzed that different types of hydrogen bonds have been proposed based on polybenzimidazole polymer chains, phosphoric acid networks, and imidazole rings from ZIF, which helps protons to jump from one proton-carrier (ZIF surface) site to adjacent proton-carrier site (next neighboring ZIF). Therefore, the proton conductivity efficiency is greatly improved in the designed PEM.

Other MOFs are also applied, while the lack of the ability to form film adds to the difficulty. But there are still successful designs, for instance, Mukhopadhyay et al. [62] synthesized aryl ether-type PBI (OPBI) composite membranes by loading post synthetically modified UiO-66-NH_2_ MOFs. They found that the proton conductivity of the composite membrane reached an extremely high value of 0.308 S cm^−1^ at 160 °C, much higher than that of pristine OPBI film (0.067 S cm^−1^ at 160 °C). Authors concluded that the hydrophilic -SO_3_H groups in the post synthetically modified MOFs help to form proton conduction channels through hydrogen-bonding interactions with PA, and more -SO_3_H groups in the composite result in more hydrophilic ion channels and contribute to more PA loads, which in turn promotes proton conduction.

Table 2 presents recent achievements in various modified PBI-based PEMs on the parameters of proton conductivity and power density. It can be seen that the proton conductivity of the PBI PEMs with multiple modifications are relatively higher than that of those with a single modification.

## 4. Progress of Modification of PBI-Based Membranes for DMFCs

### 4.1. Acid Doping in PBI Membranes for DMFCs

Because of the methanol’s excellent resistance performance, PBI membranes have also been attempted in DMFC applications [9]. However, the poor proton conductivity of pure PBI still needs to be significantly improved before applying in DMFCs. Besides, the methanol resistance performance and thermal stability of PBI films should not be spoiled. To achieve these goals, methods of doping acids are primely developed.

Acids are first applied in PBI PEMs for DMFCs in 1995 by Wainright et al. [104], they applied H_3_PO_4_-doped PBI as a membrane system and obtained a greatly lowered methanol crossover rate in comparison to Nafion. After 26 years, such issues are still in concern, the potential of acid-doped PBI PEMs for DMFC applications has been widely recognized. Key developments have been achieved as early as 1996, Wang et al. [105] applied acid-doped PBI PEM to DMFC and performed real-time analysis of the methanol crossover and poisoning effect in the cathode of the fuel cell. With this, the mechanism of methanol crossover was revealed and the accompanied CO poisoning effect caused by methanol oxidation at the cathode was promoted. As the inevitable of methanol crossover and the CO gas, DMFCs working at elevated temperatures are developed to solve the problem [21]. However, new problems appeared for acid-doped PBI membranes. Though the excellent thermal stability of PBI allows it to work at elevated temperatures, the acid leakage effect becomes serious when the membrane is in contact with water at high temperatures [106]. Since at elevated temperatures, weak bonds are easier to break, H_2_PO_4_^−^ groups are easier to release from the polymer structure, especially under the existence of water. Thus, modifications for PBI membranes for high-temperature DMFCs are mainly focused on promoting the acid retaining capacity. PA is still the most common use for PBI membranes in DMFCs, and designs of PBI-based polymers are targeted at holding more PA molecules.

Different methods have been developed to synthesize PBI membranes with higher acid doping levels. For instance, in the late 20th century, Mitsuyasu et al. [107] proposed a method of immersing PBI in a mixed solution of strong acid and methanol for doping acids. The obtained doping level is only 2.9 mol unit^−1^, and the proton conductivity is 10^−5^ S cm^−1^ at 160 °C. Later, modified solvent casting methods that are still widely used were developed. Such as adding solvents dimethylacetamide (DMAc) that make it easy to form high molecule weight polymers [108], trifluoroacetic acid (TFA), and polyphosphoric acid (PPA) that can obtain high acid doping levels [14]. For instance, in 2012, Liliana et al. [22] applied PPA in casting PBI-based membranes. Their prepared acid-doped PBI membrane (without other modifications) processes a proton conductivity of 10 mS cm^−1^ at 60 °C which is much higher than that cast in simple acid solutions, indicating a promoted acid doping level. The authors mentioned that the promoted proton conductivity is probably due to the different pretreatment methods, but unfortunately, no detailed mechanisms were presented. Qian et al. [109] also applied PPA in preparing the PBI membrane and compared it with that prepared by the conventional DMAc process. Authors presented that higher acid doping levels can be obtained by the PPA process since the formation of physical gel, leading to higher proton conductivity. Recently, with the development of technology, the casted acid-doped PBI membranes for DMFCs could have higher acid doping levels and better stability. As reported by Cheng et al. [110], the prepared PA doped PBI membrane exhibits a proton conductivity of 41 mS cm^−1^ at 200 °C and the PA doping level reached 7.2 PA per polymer unit. The prepared membrane has also shown considerable stability with silica loaded. Authors attribute the improved properties of the membrane to the formation of the PA/phosphosilicate nanocluster phase during prior polarization treatment. More details on such nanofiller are introduced in Section 4.3.

**Figure 6:**
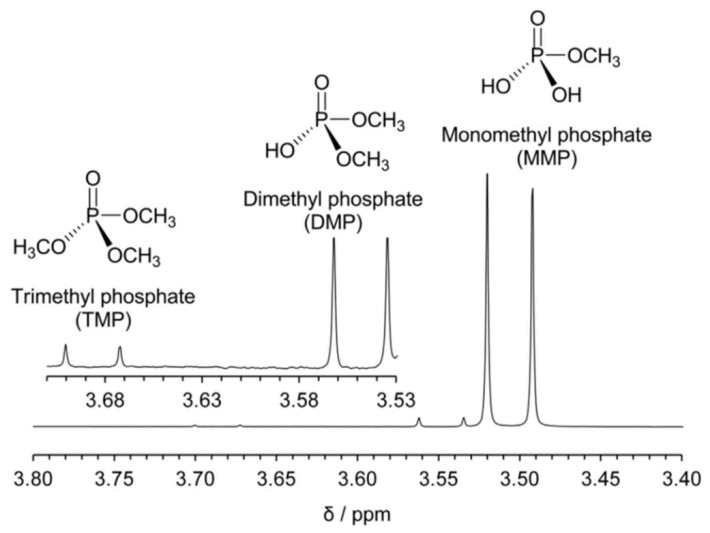
Identification of methyl phosphate at a degraded MEA and the chemical structures of the corresponding phosphoric acid methyl ester derivatives from Ref. [111].

However, there are also problems with phosphoric acid doped PBI membranes in DMFCs. Mechanisms on the degrading of acid-doped PBI PEMs in DMFCs were also promoted. Aili et al. [111] proposed that the esterification between the doped phosphoric acid and methanol is the main reason for the performance degradation of DMFCs. The aforementioned opinion was proven by the identification of methyl phosphate at a degraded MEA after a long period of working under 150 °C, as presented in Figure 6. It is suggested that the esterification could be suppressed by enhancing the water activity.

Table 3 listed the membrane properties of some representative studies on promoting the proton conductivity of PBI PEMs in DMFCs by PA doping. In addition to modifying the preparation methods of PBI PEMs, methods of synthesizing PBI with modified groups, applying nanomaterials, and using crosslinkers are also applied in promoting the acid retaining properties of PBI in high-temperature DMFCs, the relative works are introduced in the next section.

### 4.2. Modifying the Polymer Structure of PBI Membranes for DMFCs

Modifying the polymer structure can also be applied to PBI membranes in DMFCs. Besides promoting the proton conductivity, the superior nature (such as good mechanical and chemical stability in high temperature, high methanol resistance) of the original PBI should also be retained. To achieve this goal, great efforts have been made and various modifications have been applied to the main chains of PBI [13,16,113,114]. Detailed properties are presented in Table 4.

Modified PBI PEMs for DMFCs still usually work at temperatures between 60–80 °C, few of them work at above 100 °C [5] as the difficulty for gas water to wet membranes. Besides, the CO concentration in DMFCs is not as high as that in reforming gas, it is not necessary to work at a temperature that high. Since the common aim of promoting the proton conductivity, the main directions of modifications on PBI are similar to that for hydrogen-air PEMFCs, while some details should be considered for DMFCs. For instance, in 2007, Chuang et al. [113] synthesized fluorine-containing PBI and combined that with montmorillonite (MMT). The CF_3_ groups on PBI polymer units allowed more phosphoric acid adsorbed and thus improved acid doping level and promoted the proton conductivity. The additional MMT further improved the methanol resistance performance of the modified PBI and was close to that of the original m-PBI. Liliana et al. [22] applied AB-PBI and crosslinked with Nafion 117 to make up for the shortcomings for each. Compared with m-PBI, the simple structured AB-PBI (structure shown in Figure 4c) exhibited higher methanol resistance and higher proton conductivity while combined with Nafion, giving an overall improvement in the PEM performance for both PBI- and Nafion-based membranes in DMFCs.

### 4.3. Applying Inorganic Nanofillers to PBI Membranes for DMFCs

To further improve the proton conductivity of PBI PEMs for DMFCs, inorganic nanofillers can also be applied. Such methods are promising as nanomaterials are inexpensive and easy to obtain. Besides, nanofillers can also help in improving the mechanical properties of PBI PEMs. Since in elevated temperatures, PBI PEMs with high acid doping levels might be easily broken under the high gas pressure [113]. The mechanical properties should also be enhanced since the life of membranes is also an important index in actual DMFC applications. Various inorganic nanofillers have been applied and proven to be effective in promoting the properties of PBI PEMs, such as MMT [113], Graphene oxide (GO) [116], and SiO_2_ [98]. With such components, the available ion exchange sites in PBI membranes can be increased, and thus the proton conductivity is promoted [114,115,116,117,118,119,120].

For PBI PEMs used for DMFCs, doping inorganic nanofillers can add to the ion exchange sites through a variety of mechanisms, either promoting the acid retaining capacity or improving the proton transport through the hopping mechanism. It depends on the nature of materials and doping methods. For instance, Suryani et al. [69] applied sulfonated silica nanoparticles (SA-SNP) to PBI and synthesized nanocomposite films. The membrane with 10wt% SA-SNP doped exhibited highly enhanced proton conductivity that is nearly 3-fold as that of pristine PBI membranes. The methanol permeability is also as low as 3.3 × 10^−7^cm^2^ s^−1^. The authors analyzed that the sulfonic acid groups of SA-SNP can form ionic linkages with the PBI chains, which enhanced the compatibility and created more sites to hold PA groups. With this, the PA doping level of the composite membrane become higher and reached 12.3. Besides, the mechanical strength of the PBI/SA-SNP nanocomposite membrane can also be improved, making such membranes closer to fuel cell applications.

Other mechanisms for promoting the conductivity of PBI-based membranes were also reported. As presented by Kumar et al. [121], by incorporating GO-Fe_3_O_4_ nanocomposites to PBI, a PEM with high ion transport efficiency was designed. With the GO-Fe_3_O_4_ nanofillers in the PBI matrix, the ion conductivity reached 5.24 × 10^−2^ S cm^−1^, which is 2.4-fold as that of the pristine PBI electrolyte. It is explained that the steric hindrance of the Fe_3_O_4_ and GO nanofillers may prevent polymer chain alignment and reduce the crystallinity of the polymer. As a result, the free volume is flexibly polymeric and more ionic transport paths are offered. In addition, the GO hydrophilic functional groups can form hydrogen bonding and electrostatic interactions. With the two advantages for ion transport, the conductivity of the PBI membrane with GO-Fe_3_O_4_ becomes high.

### 4.4. Applying Polymer Crosslinkers to PBI Membranes for DMFCs

**Figure 7:**
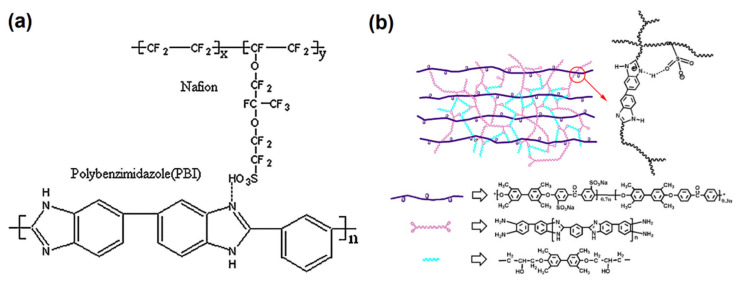
(**a**) The formation mechanism between the sulfonic acid group of Nafion and the imidazole nitrogen of PBI from Ref. [127]; (**b**)Schematic representation of the SPEEK/o-PBI/TMBP composite membranes from Ref. [125].

Both covalent and ironical crosslinking can be applied to improve the proton conductivity and reduce the methanol crossover of PEMs in DMFCs. It is well known that PBI is a type of basic polymeric material and can easily form acid-base compounds with acid polymeric materials [122]. Those are the ironical crosslinking cases, many acid polymers have been applied to form acid-base composites with PBI [30,123]. Sulfonated poly(arylene ether sulfone) (SPAES) [121], sulfonated poly(ether ether ketone)s (SPEEK) [120,124,125], sulfonated poly(imide)s (SPI) [126], and Nafion are all typical polymers with acidic groups, and their crosslinking with PBI have all been researched. As presented in Figure 7a, Ahmad et al. [127] performed the combination of PBI and Nafion, since the sulfonic acid groups of Nafion can interact with the N-base of the PBI. The membrane with the crosslinked composite polymer exhibited both high proton conductivity (nearly 20 mScm^−1^ at room temperature) and good methanol resistance performance (2.339 × 10^−7^ cm^−2^s^−1^). Compared with Nafion, the methanol permeability has been reduced to smaller than one-fourth while the proton conductivity is only 10% lower. As shown in Figure 7b, Han et al. [125] synthesized a composite polymer membrane with the crosslinking of SPEEK and o-PBI. With the interaction between the -NH groups in PBI and the -SO_3_ groups in SPEEK, a three-dimensional network polymer structure that is beneficial to proton transport formed. The PEM exhibited excellent proton conductivity performance, which is 0.14 S cm^−1^ at 80 °C, even similar to that of Nafion 117 (0.142 S cm^−1^). The methanol permeability is also as low as 2.38 × 10^−8^ cm^−2^ s^−1^, far lower than that of Nafion. Besides, the mechanical properties, thermal stability are also considerable. Such design of polymer membrane is successful and close to applications in DMFCs. 

For covalently crosslinked cases, the linkable groups should be introduced into the chain of the polymer. This can be achieved by synthesizing from cross-linkable monomers or making modifications to the original polymer. Many considerable modifications on PBI have also been performed by covalent crosslinking. As Wang et al. [128], applied sulfonated polyphosphazene (SPOP) and PBI and formed covalently crosslinked polymer structures. It is explained that the =N- groups in PBI can form covalent bonds with the -SO_3_ groups in SPOP, composing stable polymer structures. The designed membrane has shown considerable proton conductivity of 0.143 Scm^−1^ at 180 °C and low methanol permeability of 7.95 × 10^−8^ cm^−2^ s^−1^ at 60 °C. It is also reported that the performance can also be moderated by changing the doping amount of SPOP, indicating a flexible design and wide application range.

Table 5 presents recent achievements in various modified PBI-based PEMs in DMFCs on the parameters of methanol permeation, proton conductivity, and power density. Compared with the studies presented in Table 3 and Table 4. It can be seen that the properties of the PBI PEMs with the combination of different modification methods are higher than that of those single modifications.

## 5. Conclusions and Perspective

This article examined the methods to promote the proton conductivity of PBI-based PEMs in both hydrogen-air PEMFCs and DMFCs. The main advantage of PBI PEMs exists in two aspects: First, the high mechanical and chemical stability allow it to work at high temperatures that Pt-based electrode catalysts can bear higher CO concentration. Second, PBI PEMs usually process high methanol resistance which has the potential to solve the primate issue of DMFCs. While the low proton conductivity makes it hard to apply in fuel cells. Efforts are made for several decades to improve the proton conductivity and retain the good natures.

The most direct and effective method is doping acids (mainly phosphoric acid) which could significantly improve the proton conductivity of PBI PEMs. However, the limited doping level of pristine PBI, as well as the acid leakage effect, greatly reduced the life of PBI membranes. To solve these problems, additional methods of synthesizing modified structures of PBI, applying nanomaterials, using other polymer crosslinkers are proposed, and considerable effects have been obtained. Such modification methods can also bring promotions in the stability and mechanical strength of PBI membranes which makes them closer to the requirements of fuel cell applications. In recent years, with the development of synthesizing technology, methods to make specifically structured membranes such as porous PBI are designed and can further improve the proton conductivity level to a new degree. Besides, MOFs which are widely studied these days are also applied in PBI PEMs. With these, the development of PBI membranes is rapid and not far from extensive use. However, detailed mechanisms of properties improvements are still not clear, especially those with comprehensive modifications. Many studies have only presented the superior performance of the designed membrane or only explained the possible effects of the key components. Only a few of them have given credible explanations on the detailed mechanisms of the improved proton transport of the modified PBI membrane. In the future, PBI-based PEMs are expected to be meticulous in design and process higher acid doping levels with considerable mechanical properties. The mechanisms for achieving these excellent properties are also expected to be more complete. For next generation of PBI-based membranes in PEMFCs, they are expected to have higher mechanical strength and chemical stability at high temperatures with high acid doping levels. For future PBI PEMs in DMFCs, under the guarantee of competitive proton conductivity, the methanol crossover is expected to be further lowered and closer to zero.

## Figures and Tables

**Table 1:** The membrane properties of some representative studies on promoting the proton conductivity of PBI PEMs in hydrogen-air PEMFCs by acid doping.

Type of Membrane	Synthesis Method	*σ* (S·cm^−1^)	Operating Temperature (°C)	Publishing Year	Ref.
PBI/ZrP + H_3_PO_4_ PBI/SiWA + SiO_2_ + H_3_PO_4_ PBI/PWA + SiO_2_ + H_3_PO_4_	solvent casting	9 × 10^−2^ (RH = 5%) 3–4 × 10^−2^ (RH = 5%) 1.5 × 10^−3^ (RH = 5%)	200 200 150	2003	[70]
PBI/H_3_PO_4_	solvent casting	5.90 × 10^−2^ (RH = 30%)	150	2004	[55]
PBI/H_3_PO_4_ (solvent:DMAc)	solvent casting	6.1~6.3 × 10^−2^	140	2006	[34]
PBI/H_3_PO_4_	solvent casting	6.8 × 10^−2^	200	2006	[71]
PBI/H_3_PO_4_	solvent casting	0.039	190	2006	[65]
H_3_PO_4_/PMIH_2_PO_4_/PBI	solvent casting	2.0 × 10^−3^	150	2008	[55]
SPEEK/PBI/H_3_PO_4_	solvent casting	0.080	80	2008	[72]
PBI/HMI-Tf/H_3_PO_4_	solvent casting	1.6 × 10^−2^	250	2011	[17]
cPBI–BF_4_ + H_3_PO_4_	solvent casting	0.117	170	2019	[73]
QPPSf-PBI + H_3_PO_4_	solvent casting	0.09	200	2020	[74]
PBI/Mim_7_^+^/PAM_2_ + H_3_PO_4_	solvent casting	0.226	180	2021	[75]

*σ*-Proton conductivity.

**Table 2:** The membrane properties of some representative studies on PBI PEMs in hydrogen-air PEMFCs (Acid components have been omitted in the column of “type of membrane” for phosphoric acid doped cases.).

Type of Membrane	Synthesis Method	*σ* (S·cm^−1^)	Energy Density	Operating Temperature (°C)	Publishing Year	Ref.
2OH-PBI/PPA	solvent casting	0.350	0.294 W·cm^−2^	180	2009	[82]
OPBI/AMS	solvent casting	0.125	N/A	160	2011	[69]
HB-PBI	solvent casting	0.168	0.346 W·cm^−2^	150	2013	[83]
P-PBI	solvent casting	0.160	N/A	150	2010	[80]
AB-PBI	N/A	N/A	0.305 W·cm^−2^	120	2014	[81]
F_6_PBI	solvent casting	N/A	0.322 W·cm^−2^	160	2015	[23]
c-sTiO_2_-PBI-OO	solvent casting	0.098	0.356 W·cm^−2^	160	2018	[78]
Bipy-PBI	solvent casting	0.037	0.779 W·cm^−2^	120	2019	[79]
PBI/ZC-SiO_2_	solvent casting	0.190	N/A	160	2012	[26]
PPBI-SPI_25_-SCYb_x_	solvent casting	0.131	0.59 W·cm^−2^	180	2020	[63]
ILGO/PBI	hot-pressed	0.035	0.32 W·cm^−2^	175	2015	[25]
PBI-SGO	solvent casting	0.029	40% higher than pure PBI membrane	150	2020	[27]
PBI/ImGO	solvent casting	0.078	N/A	150	2021	[101]
PBI-TMBP	160 ℃ heating	0.051	N/A	200	2011	[28]
PBI/FeSPP-PWA	hot-pressed	0.110	N/A	170	2018	[89]
PBI-TGIC/SPAN	solvent casting	0.130	N/A	180	2018	[29]
NbPBI-TPAm	solvent casting	0.072	0.385 W·cm^−2^	160	2021	[90]
PSM-OPBI	solution casting	0.308	N/A	160	2020	[62]
Porous PBI	VIPS+ solvent casting	0.071	0.485 W·cm^−2^	180	2021	[33]
asymmetric PBI	soft-template	0.066	0.295 W·cm^−2^	160	2014	[32]
PBI@ZIF	solvent casting	0.091 ± 0.002	N/A	200	2018	[59]
mp-PBI	hard templating	0.011	N/A	180	2008	[61]
PBI-PBz-NF- X	electrospinning	0.17	0.67W·cm^−2^	160	2013	[96]
Spbi-*b*-PIs	Solvent evaporation	0.2	N/A	160	2018	[102]
PBI-BS	solvent casting	0.031	0.551W·cm^−2^	160	2012	[84]
PBI/IL	solvent casting	1.86	0.039 W·cm^−2^	190	2013	[97]
SPF-70	solvent casting	N/A	0.186 W·cm^−2^	120	2017	[98]
PBI/La_2_Ce_2_O_7_	solvent casting	0.093	0.43 W cm^−2^	180	2014	[86]
PPBI-SPI-SCYb	solvent casting	0.131	N/A	180	2020	[63]
PPBI-SPE-ZQD	solvent casting	0.162	0.67 W/cm^2^	180	2021	[103]

N/A-not reported. *σ*-Proton conductivity.

**Table 3:** The membrane properties of some representative studies on promoting the proton conductivity of PBI PEMs in DMFCs by PA doping.

Type of Membrane	Synthesis Method	*ρ* (cm^2^·s^−1^)	*σ* (S·cm^−1^)	Operating Temperature (℃)	Publishing Year	Ref.
90 μm H_3_PO_4_/PBI	solvent casting	5~11 mA·cm^−2^ (CO_2_)	N/A	180	1996	[105]
PBI/H_3_PO_4_	N/A	N/A	10^−5^	160	2000	[107]
mPBI/H_3_PO_4_	solvent casting	N/A	10^−4^~10^−1^	150	2015	[111]
PBI/H_3_PO_4_	N/A	5~35 mA cm^−2^	N/A	140~160	2015	[112]
PBI/H_3_PO_4_	N/A	6~26 mA cm^−2^	N/A	175	2018	[21]

*ρ*-Methanol permeability. *σ*-Proton conductivity. N/A-not reported.

**Table 4:** The membrane properties of some representative studies on promoting the proton conductivity of PBI PEMs in DMFCs by synthesizing polymer structures (Acid components have been omitted in the column of “type of membrane” for phosphoric acid doped cases.).

Type of Membrane	Synthesis Method	*ρ* (cm^2^·s^−1^)	*σ* (S·cm^−1^)	Energy Density	Operating Temperature (°C)	Publishing Year	Ref.
PBI_4N	solvent casting	2 × 10^−8^	>0.1	N/A	80	2006	[16]
fluorine-containing PBI/m-MMT	solvent casting	6.2 × 10^−9^	7.94 × 10^−5^	N/A	160	2007	[113]
PES/MS-p-PBI	solvent casting	N/A	0.072	506.9 mW cm^−2^	70	2010	[114]
SPEEK/o-PBI	solvent casting	2.38 × 10^−8^	0.14	N/A	80	2011	[13]
AB-PBI	High-temperature casting	1.63~2.07 × 10^−7^	0.041	N/A	60	2012	[22]
QOPBI-x	solvent casting	1.86 × 10^−8^	0.122	75.6 mW cm^−2^	60	2020	[115]

*ρ*-Methanol permeability. *σ*-Proton conductivity. N/A-not reported.

**Table 5:** The membrane properties of some representative studies on PBI PEMs in DMFCs (Acid components have been omitted in the column of “type of membrane” for phosphoric acid doped cases.).

Type of Membrane	Synthesis Method	*ρ* (cm^2^·s^−1^)	*σ* (S·cm^−1^)	Energy Density	Operating Temperature (°C)	Publishing Year	Ref.
PBI/ZC-GO	solvent casting	1.38 × 10^−7^	1.83 × 10^−2^	N/A	25–90	2015	[116]
PBI/SiO_2_	casting & pre-heat treatment	N/A	2.9–4.1 × 10^−2^	237 mW cm^−2^ (260 °C)	200–250	2020	[110]
PBI/SA-SNP	polycondensation reaction	3.3 × 10^−7^	N/A	N/A	N/A	2009	[69]
PBI/GO-Fe_3_O_4_	solvothermal	9.6 × 10^−7^	4.6 × 10^−2^	233 mW cm^−2^	80	2020	[121]
Nafion-PBI-ZP	solvent casting	2.34 × 10^−7^	0.020	N/A	room temperature	2011	[127]
(PBI)/ZC-PAMAM	solvent casting	5.23 × 10^−8^	1.83 × 10^−2^	N/A	80	2013	[30]
M-SPVdF-co-HFP/PBI	solution polymerization	1.22 × 10^−6^~9.71 × 10^−7^	0.0042- 0.0301	36–39 mW cm^−2^	60–90	2015	[123]
SPAES-PBI	in situ polymerization	2.15 × 10^−7^	0.077	N/A	30	2010	[124]
SPPO-PBI	catalyst painting technique	5.5 × 10^−7^	0.012	57.6 mW cm^−2^	25–70	2011	[126]
SPOP/PBI	solvent casting	1 × 10^−7^~2 × 10^−6^	0.005–0.08	N/A	60	2005	[129]
SPEEK/PBI	solvent casting	N/A	0.0046	N/A	60	2008	[124]
SPEEK/o-PBI/TMBP	solvent casting	2.38 × 10^−8^	0.14	N/A	80	2011	[125]
N/PVFP-BI	Electrospinning process	1.88 ± 0.02 × 10^−8^	1.3 ± 0.1 × 10^−2^	84.6–106.2 mW cm^−2^	70–90	2014	[130]
sPEEK/ZrPh/ PBI	solvent casting	N/A	0.0294	N/A	25	2005	[117]
sPEEK/ZrPh/ PBI	solvent casting	4.7 × 10^−7^	18.2 × 10^−2^	N/A	110	2005	[120]

*ρ*-Methanol permeability. *σ*-Proton conductivity. N/A-not reported.

## Data Availability

Not applicable.
